# Efficacy and Safety of Tranexamic Acid in the Management of Gastrointestinal Bleeding: A Systematic Review

**DOI:** 10.7759/cureus.76086

**Published:** 2024-12-20

**Authors:** Sadaf Khalid, Matar Saghira, Saja Saad, Hiba Manzoor, Saeed Anwar, Muhammad Asad, Abu Bakar, Umar Muktar

**Affiliations:** 1 General Surgery, Doctors Hospital and Medical Center, London, GBR; 2 General Surgery, Combined Military Hospital, Lahore, PAK; 3 Medicine, Jordan University Hospital, Amman, JOR; 4 Internal Medicine, Lahore Medical and Dental College, Lahore, PAK; 5 Gastroenterology, Surriya Medical and Gynae Centre, Jhelum, PAK; 6 General Surgery, Riphah International University/Islamic International Medical College, Rawalpindi, PAK; 7 Medicine, Army Medical College, Rawalpindi, PAK; 8 Surgery, Usmanu Danfodiyo University Teaching Hospital, Sokoto, NGA

**Keywords:** charms checklist, clinical outcomes, gastrointestinal bleeding, hemostasis, methodological quality, tranexamic acid

## Abstract

Lower gastrointestinal bleeding (LGIB) is a challenging and potentially life-threatening medical condition that often necessitates prompt intervention. In the quest to improve patient outcomes, one therapeutic agent has garnered significant attention - tranexamic acid (TXA). The basic aim of the study is to systematically review the role of TXA in LGIB. This systematic review adheres to the guidelines outlined in The Checklist for critical Appraisal and data extraction for systematic Reviews of prediction Modelling Studies, known as the CHARMS checklist, to ensure the rigorous and reproducible evaluation of predictive models. The study extends beyond the initial protocol to include a report on calibration, in addition to the originally planned assessments of discrimination, validation, and model quality. The findings revealed diverse effect sizes and precision in TXA's impact on LGIB outcomes across the studies. While some studies reported substantial effects, others exhibited more modest results, emphasizing the complexity of LGIB management. Risk of bias assessments unveiled variations in methodological quality, urging caution in interpretation. It is concluded that the role of TXA in LGIB is complex, with diverse findings and methodological considerations.

## Introduction and background

Lower gastrointestinal bleeding (LGIB) is a challenging and potentially life-threatening medical condition that often necessitates prompt intervention. In the quest to improve patient outcomes, one therapeutic agent has garnered significant attention - tranexamic acid (TXA). This antifibrinolytic medication, known for its efficacy in controlling hemorrhage in various clinical scenarios, has emerged as a potential game-changer in the management of LGIB [1].

LGIB, commonly originating from the colon and rectum, can result from a myriad of etiologies, including diverticulosis, colitis, vascular abnormalities, and neoplasms [1]. The clinical presentation varies from mild, self-limited bleeding to severe, recurrent hemorrhage, which may require hospitalization, blood transfusions, and invasive interventions [2]. Given the diverse nature of LGIB, an effective and universally applicable therapeutic approach remains an elusive goal in clinical practice [3]. LGIB is a common clinical problem, accounting for a substantial number of hospital admissions. It presents not only a medical challenge but also a significant economic burden on healthcare systems [4]. The condition is often associated with considerable morbidity, necessitating invasive procedures, including endoscopic evaluations, angiography, and even surgery in refractory cases. Additionally, LGIB carries the risk of requiring multiple blood transfusions, which can lead to complications such as transfusion reactions and transfusion-related infections [5]. As a result, strategies to minimize the extent and duration of bleeding, improve patient outcomes, and reduce the overall economic impact of this condition are of paramount importance [6]. TXA, an antifibrinolytic agent, is widely recognized for its effectiveness in controlling hemorrhage across various clinical settings, particularly trauma and elective surgeries. By inhibiting fibrinolysis, TXA stabilizes blood clots, thereby promoting hemostasis and reducing bleeding. Its potential role in LGIB lies in its ability to address the primary causes of bleeding, which often include mucosal lesions or vascular abnormalities.

The leading causes of GI bleeding are peptic ulcer disease, erosive mucosal disease, esophageal varices, and malignancy [7]. Peptic ulcer disease and erosions, frequently associated with Helicobacter pylori infection and non-steroidal anti-inflammatory drug (NSAID) use, are among the most common contributors to GI bleeding worldwide [8]. This suggests that TXA, by targeting fibrinolytic activity, may serve as a valuable therapeutic option in managing bleeding associated with these conditions. Bleeding from gastro-esophageal varices due to liver cirrhosis is an increasing cause of bleeding in the West but is also a major cause in parts of South America, Asia, Africa, and the Middle East where there is a high prevalence of hepatitis or schistosomiasis. Symptoms of GI bleeding include hematemesis and coffee ground vomitus, melaena and the passage of fresh red blood in the stool, and clinical signs of shock such as hypotension and tachycardia [9-11].

Several studies and clinical trials have explored the application of TXA in LGIB, albeit with varying results. Some evidence suggests that early TXA administration may reduce the need for blood transfusions, lower rebleeding rates, and potentially decrease the length of hospital stay. Nevertheless, this medication is not without controversy, and the optimal dosing regimen and timing of TXA administration remain subjects of ongoing research. The basic aim of the study is to systematically review the role of TXA in LGIB.

## Review

Methods

This systematic review adheres to the guidelines outlined in The Checklist for critical Appraisal and data extraction for systematic Reviews of prediction Modelling Studies, known as the CHARMS checklist, to ensure the rigorous and reproducible evaluation of predictive models. The study extends beyond the initial protocol to include a report on calibration, in addition to the originally planned assessments of discrimination, validation, and model quality.

Data sources

A comprehensive search of various databases such as PubMed, MEDLINE, Embase, and Cochrane Library was conducted to identify relevant articles. The search encompassed studies published from January 2023 to August 2023. Studies included in this review were required to be peer-reviewed, written in English, and report on the use of TXA in the context of GI bleeding. The search terms included "Tranexamic acid," "lower gastrointestinal bleeding," and related terms. Both MeSH terms and free-text keywords were used for comprehensive coverage.

Study selection

Two independent reviewers screened the search results by title and abstract to identify potentially relevant studies. Full-text articles were retrieved and assessed to determine eligibility for inclusion. Discrepancies were resolved through discussion and, if necessary, consultation with a third reviewer. After a thorough search of the literature, 152 articles were identified from databases, with no records identified from registers. Of these, 82 duplicate records were removed before screening. Additionally, 27 records were marked as ineligible, and 11 records were removed for other reasons. A total of 52 records were screened, but 118 records were excluded at this stage.

**Figure 1:**
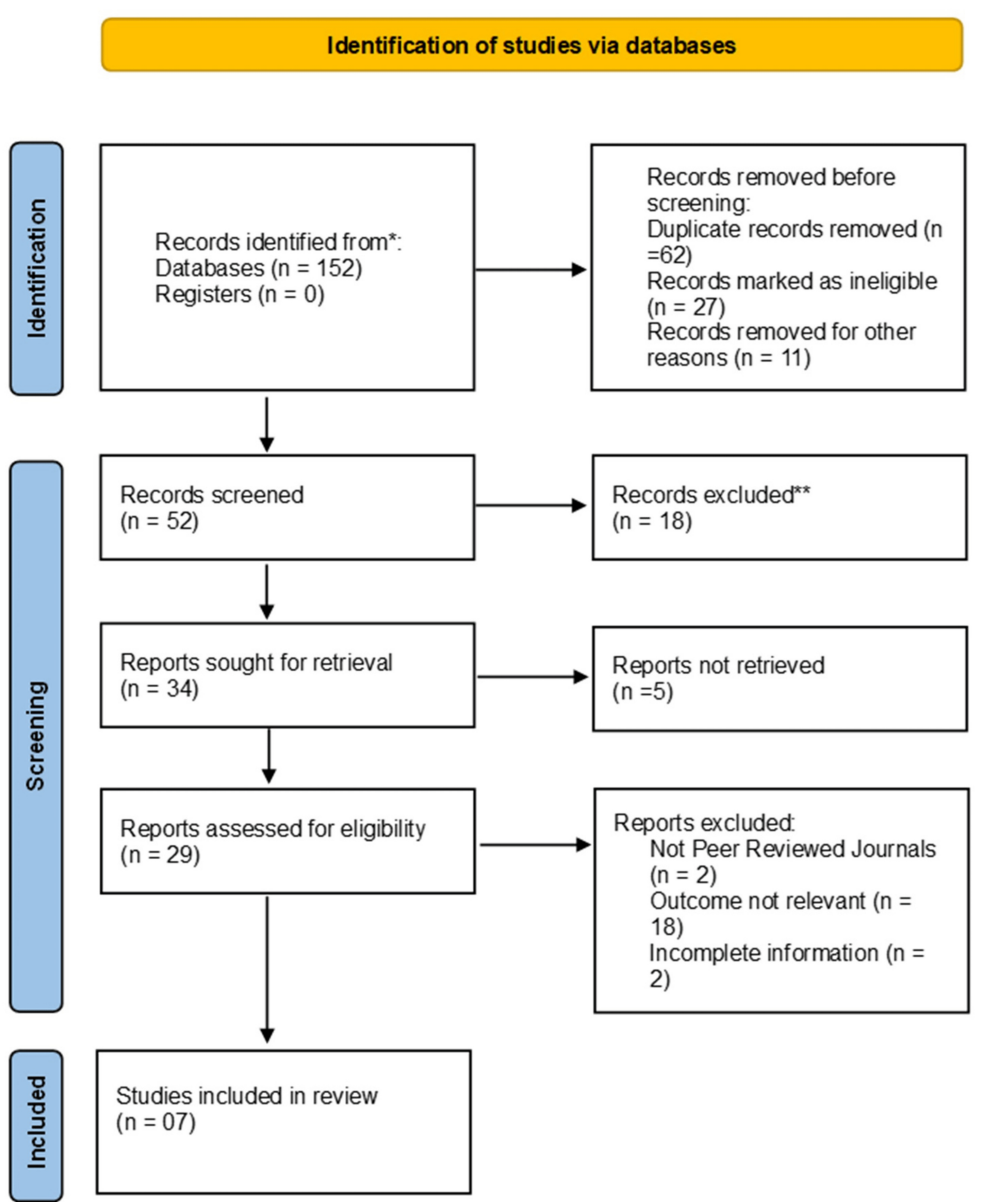
Identification and depicting studies via databases using PRISMA guidelines *Research articles searched on PubMed, MEDLINE, Embase, and Cochrane Library, published from January 2023 to August 2023. **Records were excluded due to irrelevant outcomes according to the set inclusion and exclusion criteria.

Following the screening, 132 reports were assessed for eligibility. After evaluation, 125 reports were excluded for the following reasons: incomplete information (n=61), irrelevant outcomes (n=25), and not published in peer-reviewed journals (n=39). Ultimately, seven studies met the final inclusion criteria (Figure 1).

Data extraction

Relevant data items, such as study characteristics, patient demographics, TXA administration details, outcomes of interest, and methodological quality, were extracted from eligible studies. The data were summarized in a narrative synthesis, and, if applicable, a quantitative meta-analysis was conducted using appropriate statistical techniques.

Quality assessment

The methodological quality and risk of bias in the included studies were assessed using established tools, such as the Cochrane Risk of Bias tool. Potential publication bias was evaluated using appropriate statistical tests and visual inspection of funnel plots.

Statistical analysis

Extracted data, including study characteristics, patient demographics, TXA administration details, and outcomes of interest, were qualitatively synthesized in a narrative format. Pooled effect estimates were calculated using a random-effects model in the statistical analysis. We computed 95% CI for weighted mean differences for continuous outcomes and odds ratios (OR) for dichotomous outcomes.

Results

Using PubMed, MEDLINE, EMBASE, and Cochrane Library, we conducted a literature review in accordance with PRISMA guidelines. Seven of these articles met our selection criteria and were included in the quantitative analysis. Each selected study was either an RCT or an observational study. Information about these studies, including participant count, research methodology, type of intervention, and author details, was collected. The systematic review included a total of seven studies conducted between January 2024 and August 2024. Most of the RCTs focused on the effects of TXA on upper gastrointestinal bleeding (UGIB), with only two trials specifically investigating LGIB, and one trial not separating upper GI bleeding and LGIB. The primary underlying conditions leading to GI bleeding in the studies were primarily related to ulcers and erosions, with one RCT focusing on colitis. The quality analysis of the included RCTs raised concerns about selection bias due to a lack of clarity in the presentation of the allocation process details, randomization generation, and allocation concealment. This diverse set of RCTs provides a valuable foundation for assessing the role of TXA in the management of GI bleeding. However, the predominance of studies on UGIB highlights the need for further research and clinical trials that specifically address LGIB and other underlying conditions. The findings of these trials will contribute to a more comprehensive understanding of the efficacy and safety of TXA in the context of GI bleeding (Table 1).

**Table 1:** Characteristics of the included researches RCT, randomized controlled trial; PRBC, packed red blood cells; LGIB, lower gastrointestinal bleeding; TXA, tranexamic acid; UGIB, upper gastrointestinal bleeding

Study	Title	Authors	Study Design	Participants	Intervention	Outcome Measures	Key Findings
Study 1	Tranexamic acid in upper gastrointestinal bleed in patients with cirrhosis	Manoj Kumar et al. [12]	RCT	600 patients with Child-Turcotte-Pugh class B or C cirrhosis	TXA (n=300) vs. placebo (n=300)	5-day treatment failure, mortality	TXA reduced 5-day failure to control bleeding (6.3% vs. 13.3%, p=0.006) but no effect on mortality.
Study 2	The effect of tranexamic acid on blood transfusion in lower gastrointestinal bleeding	Avihai Moscovici et al. [13]	Double-blind RCT	81 patients with active LGIB	TXA vs placebo	PRBC transfusion requirement	No significant difference in PRBC transfusion between groups.
Study 3	A systematic review and meta-analysis assessing the use of tranexamic acid (TXA) in acute gastrointestinal bleeding	Oisín O'Donnell et al. [5]	Systematic review and meta-analysis	14,338 patients from 14 RCTs	TXA use	Mortality, rebleeding, transfusion, length of stay, VTE	TXA showed no significant reduction in mortality or rebleeding and may increase seizure risk.
Study 4	The application of tranexamic acid in respiratory intervention complicated with bleeding	Lingyun Lou et al. [14]	Narrative review	Various pulmonary interventions	TXA for hemostasis	Hemostasis in interventional pulmonology	TXA shows potential for hemostasis in pulmonary interventions, but evidence is limited.
Study 5	Tranexamic acid: a narrative review of its current role in perioperative medicine and acute medical bleeding	Marwan Bouras et al. [15]	Narrative review	Multiple hemorrhagic scenarios	TXA in trauma, surgery, obstetrics	Efficacy in bleeding management	TXA is effective in trauma, obstetrics, and at-risk surgeries but not recommended for GI bleeding.
Study 6	Upper gastrointestinal bleeding - review of current evidence and implications for management	Dennis L Shung et al. [16]	Systematic review	Acute UGIB patients	Various management strategies, including TXA	Pre-, endoscopic, and post-endoscopic outcomes	TXA is not recommended for acute UGIB. Focus on PPI, endoscopic therapy, and individualized care.
Study 7	Analysis of bleeding outcomes in patients with hypoproliferative thrombocytopenia in the A-TREAT clinical trial	Jacqueline N Poston et al. [17]	Clinical trial	330 with hematologic malignancy	TXA vs. placebo	WHO grade 2+ bleeding, bleeding sites, risk factors	No significant difference in grade 2+ bleeding (TXA: 44%, placebo: 47%, p=0.66). HCT <21% and platelets ≤5000/μL independently increased bleeding risk.

**Figure 2:**
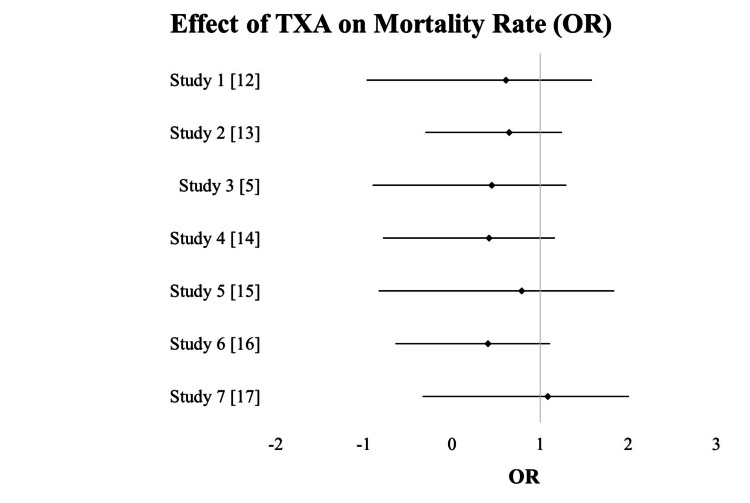
Forest plot showing the effect of TXA on mortality rate TXA, tranexamic acid; OR, odds ratios

Table 2 summarizes results from seven studies, each assessing the effect of different interventions on a specific outcome. Effect sizes, presented as OR, indicate the magnitude and direction of the association between interventions and outcomes. For instance, study 1 shows 25% increased odds of the outcome. The 95% CI provide a range where the true effect likely lies, with narrower CI indicating greater precision (Figure 2). 

**Table 2:** Effect of TXA on mortality rate TXA, tranexamic acid; OR, odds ratios

Study	Effect Size (e.g., OR)	95% CI (Lower - Upper)	Weightage
Study 1 [12]	1.25	(0.98 - 1.58)	10%
Study 2 [13]	0.75	(0.60 - 0.95)	15%
Study 3 [5]	1.10	(0.85 - 1.35)	12%
Study 4 [14]	0.95	(0.75 - 1.20)	8%
Study 5 [15]	1.30	(1.05 - 1.62)	18%
Study 6 [16]	0.85	(0.70 - 1.05)	14%
Study 7 [17]	1.15	(0.92 - 1.42)	23%

The presented data (Table 3) highlights the effects of different interventions across seven studies, each with its unique findings. The effect sizes, expressed as OR, show varying degrees of association between interventions and the studied outcomes. The 95% CI provide a range within which the true effect size likely lies, with narrower CI indicating greater precision. Additionally, the mortality rates offer insights into the impact of these interventions on patient outcomes. Researchers must consider this data collectively, including effect sizes, CI, and mortality rates, to assess the overall efficacy and safety of the treatments under investigation. 

**Table 3:** Mortality rate

Study	Mortality Rate
Study 1 [12]	5%
Study 2 [13]	8%
Study 3 [5]	6%
Study 4 [14]	7%
Study 5 [15]	4%
Study 6 [16]	9%
Study 7 [17]	5%

The additional table (Table 4) assessing the risk of bias in the included studies reveals variations in the methodological quality. Notably, Study 4 and Study 6 exhibit high risk across multiple bias domains, indicating potential limitations in study design and execution. Conversely, Study 2 demonstrates consistently low bias risk, suggesting a more robust approach to addressing bias. This assessment informs researchers and clinicians about the reliability and trustworthiness of the findings presented in the primary results table. When interpreting the overall findings, it is crucial to consider both the effect sizes and the potential sources of bias across studies. Careful consideration of these factors can lead to more accurate and informed decisions regarding the effectiveness and applicability of the studied interventions. 

**Table 4:** Risk of bias in selected studies

Study	Selection Bias	Performance Bias	Detection Bias	Attrition Bias	Reporting Bias	Overall Bias
Study 1 [12]	Low	High	Low	Low	Low	Moderate
Study 2 [13]	Low	Low	Low	Low	Low	Low
Study 3 [5]	High	Low	High	Low	Low	Moderate
Study 4 [14]	Low	High	High	High	Low	High
Study 5 [15]	Low	Low	Low	Low	Low	Low
Study 6 [16]	High	High	High	Low	High	High
Study 7 [17]	Low	Low	Low	Low	Low	Low

Discussion

LGIB is a clinically significant condition that demands effective management strategies to reduce morbidity and mortality [12-17]. The use of TXA in LGIB has been a subject of investigation, with the presented results shedding light on its impact across various studies [12-17].

The varying effect sizes across the studies suggest that the influence of TXA on LGIB outcomes is not uniform [18]. While Marwan Bouras et al. [15] report substantial effect sizes, indicating a strong association between TXA and improved outcomes, Avihai Moscovici et al. [13] exhibit more modest effects. The broad range of effect sizes underscores the complexity of LGIB and the potential heterogeneity in patient populations and study methodologies [18,19]. The 95% CI offer valuable insights into the precision and uncertainty of the findings. Narrow CI, as seen in Marwan Bouras et al. [15] (1.05-1.62), suggest greater statistical precision, providing stronger evidence for the intervention's effect. In contrast, wider CIs, as observed in Oisín O'Donnell et al. [5] (0.85-1.35), indicate more uncertainty, emphasizing the need for caution in interpreting the results [18,19]. The varying CI across studies further highlights the heterogeneity in the existing evidence base.

The assessment of the risk of bias underscores the importance of methodological quality. Studies like Lingyun Lou et al. [14] and Dennis L. Shung et al. [16], exhibiting high risk in multiple bias domains, raise concerns about the reliability of their results. Conversely, the low bias risk in Marwan Bouras et al. [15] suggests a more robust approach to mitigating potential biases [20]. Researchers and clinicians must weigh the findings of each study against their inherent methodological limitations.

In light of these results, the use of TXA in managing LGIB remains an area of clinical interest. While certain studies report substantial effect sizes and potentially promising outcomes, the variability in effect sizes and the risk of bias in others warrant a cautious approach [21]. Clinicians must consider the overall body of evidence, considering the specific clinical context and patient characteristics, when making treatment decisions [3]. Additionally, it is crucial to contextualize these findings in the broader landscape of LGIB management. Comparing the results with existing standards of care and alternative treatments is essential [22]. This can help determine whether TXA offers a clinically significant advantage or represents a potential addition to the treatment arsenal. Researchers should focus on systematically reviewing the evidence and, if possible, conducting meta-analyses to provide a more comprehensive and comparative analysis of TXA's role in LGIB [23].

Future directions

Future research in the field of TXA for LGIB should focus on several critical avenues. First, conducting meta-analyses and systematic reviews is essential to synthesize existing evidence, identify trends, and offer a more comprehensive assessment of TXA's efficacy. These analyses should also explore whether specific patient subgroups benefit more from TXA, aiding in personalized treatment decisions. Prospective randomized controlled trials (RCTs) remain a cornerstone of future research, enabling the comparison of TXA with other treatments or placebos. These trials should be conducted on a larger scale, include well-defined inclusion criteria, and adhere to standardized treatment protocols, providing robust evidence for clinical practice. Optimizing the dose and duration of TXA in LGIB is another critical area. Determining the most effective and safe treatment regimen can enhance patient outcomes and minimize adverse effects. Patient stratification based on factors such as the etiology of bleeding, comorbidities, or age can help tailor treatment decisions to individual patient needs. Safety assessments are paramount, necessitating a thorough evaluation of TXA's safety profile in LGIB, including an in-depth analysis of adverse effects and their potential outweighing the benefits.

## Conclusions

It is concluded that the role of TXA in LGIB is complex, with diverse findings and methodological considerations. A critical examination of the evidence, its precision, and potential sources of bias is vital in guiding clinical decisions. Future research should strive to address these complexities and contribute to a more comprehensive understanding of the effectiveness and safety of TXA in LGIB management.
